# Supplementary material for: Robust averaging protects decisions from noise in neural computations
Source: PLoS Comput Biol. 2017 Aug 25;13(8):e1005723. doi: 10.1371/journal.pcbi.1005723 (PMC5589265; doi:10.1371/journal.pcbi.1005723)
Supplement: S1 Table — (DOCX) [file pcbi.1005723.s001.docx]

| Variables | d.f. | F values | *p*-value |
| --- | --- | --- | --- |
| Mean | 20 | 99.37 | *p* < 0.001 |
| Variance |  | 292.59 | *p* < 0.001 |
| Session |  | 108.41 | *p* < 0.001 |
| Mean x Variance |  | 39.2 | *p* < 0.001 |
| Mean x Session |  | 4.14 | *p* = 0.55 |
| Variance x Session |  | 2.23 | *p* = 0.15 |
| Mean x Variance x Session |  | 4.57 | *p* = 0.045 |
